# Vasomotor action of androgens in the mesenteric artery of hypertensive rats. Role of perivascular innervation

**DOI:** 10.1371/journal.pone.0246254

**Published:** 2021-02-02

**Authors:** Lucía Isidoro-García, Diva M. Villalpando, Mercedes Ferrer

**Affiliations:** 1 Departamento de Fisiología, Facultad de Medicina, Universidad Autónoma de Madrid, Spain; 2 Instituto de Investigación Hospital Universitario La Paz (IdiPAZ), Madrid, Spain; Max Delbruck Centrum fur Molekulare Medizin Berlin Buch, GERMANY

## Abstract

Androgens may exert cardiovascular protective actions by regulating the release and function of different vascular factors. In addition, testosterone (TES) and its 5-reduced metabolites, 5α- and 5β-dihydrotestosterone (5α- and 5β-DHT) induce vasorelaxant and hypotensive effects. Furthermore, hypertension has been reported to alter the release and function of the neurotransmitters nitric oxide (NO), calcitonin gene-related peptide (CGRP) and noradrenaline (NA). Since the mesenteric arteries possess a dense perivascular innervation and significantly regulate total peripheral vascular resistance, the objective of this study was to analyze the effect of TES, 5α- and 5β-DHT on the neurogenic release and vasomotor function of NO, CGRP and NA. For this purpose, the superior mesenteric artery from male spontaneously hypertensive rats (SHR) and normotensive Wistar Kyoto (WKY) rats was used to analyze: (i) the effect of androgens (10 nM, incubated for 30 min) on the neurogenic release of NO, CGRP and NA and (ii) the vasoconstrictor-response to NA and the vasodilator responses to the NO donor, sodium nitroprusside (SNP) and exogenous CGRP. The results showed that TES, 5α- or 5β-DHT did not modify the release of NO, CGRP or NA induced by electrical field stimulation (EFS) in the arteries of SHR; however, in the arteries of WKY rats androgens only caused an increase in EFS-induced NO release. Moreover, TES, and especially 5β-DHT, increased the vasodilator response induced by SNP and CGRP in the arteries of SHR. These findings could be contributing to the hypotensive/antihypertensive efficacy of 5β-DHT previously described in conscious SHR and WKY rats, pointing to 5β- DHT as a potential drug for the treatment of hypertension.

## Introduction

It is a well-known fact that male sex steroids are involved in the maintenance of vascular function/structure [1–6] and in the regulation of blood pressure [5, 7, 8]. In addition, epidemiological studies have shown a correlation between low plasma testosterone (TES) levels and hypertension in men [9–12]. Likewise, clinical studies have reported beneficial effects of TES therapy in men with a history of cardiovascular disease [13–15], pointing to androgens as physiological modulators of blood pressure.

In recent times, more attention is being paid to the 5-dihydroreduced metabolites of TES. Different experimental findings have shown that TES and its 5-reduced dihydrometabolites, 5α- and 5β-dihydrotestosterone (5α- and 5β-DHT), produced an acute non-genomically mediated vasorelaxation of isolated blood vessels [16–18] which, in turn, could result in systemic hypotensive effects in vivo [7, 8, 19].

Additionally, hypertension is a complex multifactorial disease that involves a serious cardiovascular risk factor, and it is the leading cause of morbidity and mortality worldwide [20, 21]. This pathology is associated with vascular remodeling which can lead to increasing vascular resistance. In this respect, the mesenteric arterial bed is of particular relevance because it contributes greatly to the regulation of systemic arterial blood pressure [22]. The mesenteric artery possesses a dense perivascular innervation which releases different neurotransmitters when it is electrically stimulated, such as noradrenaline (NA), nitric oxide (NO) or the calcitonin gene-related peptide (CGRP) [23–26] which, in turn, they are involved in the regulation of vascular tone.

Strikingly, although many studies have analyzed the vasorelaxant and antihypertensive actions of TES, 5α- and 5β-DHT [7, 8, 18, 19], no studies explore in a comparative manner the effect of these androgens on the release and function of the neurotransmitters NO, CGRP and NA in the mesenteric artery of hypertensive rats. Therefore, further research in this respect could contribute to a better understanding of the mechanisms involved in the androgen-induced antihypertensive effect observed in conscious hypertensive rats [7]. In addition, previous studies have reported the ability of endogenous androgens to regulate the release and function of different factors in the arteries of normotensive rats [1–6, 27–29], which over prolonged periods of time could contribute to the development of hypertension [6]. The joint consideration of these data led us to hypothesize that androgens could modulate the release and function of the neurotransmitters NO, CGRP and NA in the mesenteric artery, and that the modulatory effect of androgens might differ between the arteries of hypertensive or normotensive rats.

Therefore, the present study aims to investigate the effect of TES and its 5-reduced metabolites, 5α- and 5β-DHT, on the release and vasomotor effect of NO, CGRP and NA in the mesenteric artery of spontaneously hypertensive rats (SHR) and Wistar Kyoto (WKY) normotensive rats.

## Materials and methods

### Animals and vascular tissue preparation

Male SHR and WKY rats, 5 months old, were obtained from the Animal Facility of the Universidad Autónoma de Madrid (UAM) (Registration number EX-021U). All the animal protocols were approved by the Research Ethics Committee of the UAM according to directives 609/86 CEE and R.D. 233/88 of the *Ministerio de Agricultura*, *Pesca y Alimentación* of Spain (PROEX 202/16). The experiments were carried out in accordance with the published Guiding Principles in the Care and Use of Animals approved by the European Union directives 63/2010 UE and Spanish regulation RD53/2013. The age of the animals was chosen because it was the same as that used in our previous studies [7, 18]. Systolic blood pressure was determined by tail-cuff plethysmography. The average systolic blood pressure of the animals (means ± SEM) was 166 ± 4 mmHg in SHR (n = 12) and 122 ± 6 mmHg in WKY (n = 14) rats; *p* < 0.05. Rats were sacrificed by inhalation of CO_2_ and subsequent decapitation; the first branch of the mesenteric artery was carefully dissected and placed in a Krebs- Henseleit solution (KHS) at 4°C containing, in mM: NaCl 115, CaCl2 2.5, KCl 4.6, KH2PO4 1.2, MgSO4 1.2, NaHCO3 25, glucose 11.1. The mesenteric artery was cleaned of adherent adipose and connective tissues and cut into 4 mm long rings.

### Analysis of neurotransmitters release

The release of the neurotransmitters NO, CGRP and NA was measured respectively using the fluorescent probe 4,5-diaminofluorescein (DAF-2), the enzyme immunoassay (EIA) kit for CGRP (SPI-BIO, Massy, France) or NA (Labor Diagnostika Nord, Nordhorn, Germany). The mesenteric arterial rings were immersed for 45 min in 10 mL of KHS at 37°C gassed continuously with a mixture of 95% O2-5% CO2 (stabilization period). Subsequently, the mesenteric arterial rings from SHR or WKY rats were divided into four groups, as follows: TES group: arteries incubated with 10 nM TES for 30 min; 5α-DHT group: arteries incubated with 10 nM 5α-DHT for 30 min; 5β-DHT group: arteries incubated with 10 nM 5β-DHT for 30 min; control group: arteries incubated with the equivalent ethanol (ETOH) volume for the androgen concentration used as vehicle, never exceeding 0.1% (v/v). Subsequently, the arterial rings were transferred to a bath containing 500 μL of KHS at 37°C and subjected to the specific period of incubation with androgens or ETOH. After three 10-min incubation periods, the medium was collected, immediately frozen and stored at -80°C to measure the basal neurotransmitter release of CGRP or NA. Regarding the determination of NO release, after the stabilization period, the arterial rings were incubated with the specific NO DAF-2 probe (0.5 μM) for 45 min, as previously described [3, 7, 8]. Once the 500 μL baths were refilled, each with its respective medium, 30-second cumulative EFS periods at 1, 2, 4, 8 and 16 Hz were applied at 1 min intervals, with two parallel platinum electrodes connected to an electro stimulator (Grass, model S44). The medium was collected, frozen and stored at -80°C to measure EFS-induced neurotransmitters release.

To determine basal and EFS-induced release of CGRP or NA, the respective EIA kits were performed following the manufacturer’s instructions as previously reported [5, 6, 30]. To determine the basal and EFS-induced NO release, the fluorescence of the medium was measured at room temperature with the use of a spectrofluorimeter (LS50 Perkin Elmer Instruments, FL WINLAB Software) at an excitation wavelength of 492 nm and an emission wavelength of 515 nm. The results were expressed as the ratio between the EFS-induced release and the respective basal release.

### Vascular reactivity study

The method used for isometric tension recording has been previously described [1–5, 18]. Briefly, the mesenteric arterial rings were suspended horizontally in an organ bath containing 5 mL of KHS at 37°C, continuously bubbled with a mixture of 95% O2-5% CO2 (pH 7.4). Two parallel stainless steel pins were inserted along the lumen of the vascular segment: one attached to the organ bath wall and the other connected to a force transducer (Grass FTO3C; Grass Instruments Co., Quincy, MA, USA), which in turn was connected to a model 7D Grass polygraph. The segments were subjected to a tension of 0.5 g which was readjusted every 15 min for an equilibration period of 90 min before performing the experiments. During this time, the bath solution was replaced with fresh KHS at 37°C every 20 min. After the equilibration period, the vessels were exposed to a high potassium solution (KCl 60 mM) to check their functional integrity. Exposure to 60 mM KCl was repeated twice to verify the reproducibility of the tissue response. Following a washout period, vascular endothelial viability was determined according to the ability of 20 μM acetylcholine (ACh) to relax precontracted segments with 0.1 μM NA. Only vascular rings in which ACh-induced relaxation was greater than 70% were used as preparations with intact endothelium. The vascular rings were immediately washed three times with KHS to recover the baseline tension.

To investigate the effect of TES, 5α-DHT, 5β-DHT on the vasomotor effect of released neurotransmitters (NO, CGRP and NA), separate mesenteric segments -from SHR or WKY rats- were incubated with each androgen (10 nM) for 30 min before performing cumulative concentration-response curves to the NO donor sodium nitroprusside (SNP, 1 pM—10 μM), exogenous CGRP (1 pM—0.1 μM) or exogenous NA (1 nM—10 μM). As explained for the neurotransmitter release experiments, a separate group of vessels was used as the vehicle control group in which the possible effect of ETOH was tested.

### Reagents

The following compounds were obtained from Sigma (St. Louis, MO, U.S.A.): testosterone (TES; 17β-hydroxy-4-andosten-3-one), 5α-DHT (17β-hydroxy-5α- androstan-3-one), acetylcholine chloride (ACh), L-Norepinephrine hydrochloride (NA), sodium nitroprusside (SNP), caltinonin gene related peptide (CGRP). 5β-DHT (17β- hydroxy-5β-androstan-3-one) was obtained from Steraloids (Newport, RI, U.S.A.).

The androgens were prepared separately as a stock solution (0.1 M) in absolute ethanol and then diluted in absolute ethanol to the concentration required for each experiment; final ethanol concentration in the tissue baths never exceeded 0.1% (v/v) of the vehicle. The remaining drugs were dissolved in distilled water except the NA which was dissolved in a NaCl (0.9%)-ascorbic acid (0.01% w/v) solution; appropriate dilutions were prepared in KHS on the day of the experiment. Moreover, SNP was kept in the dark until use to avoid light-induced degradation.

### Statistical analysis

All data were expressed as mean ± SEM; *n* indicates the number of animals used in each group. The vasoconstrictor response generated by NA was expressed as a percentage of the contraction induced by 60 mM KCl. Vasodilator responses to SNP and CGRP were expressed as percentage of inhibition of the contraction induced by NA. Differences were analyzed by means of a two-way ANOVA with Tukey´s post-hoc test to identify differences between two means. Significance was accepted at *p* < 0.05.

## Results

### Effect of androgens on the release of NO, CGRP and NA

**Fig 1:**
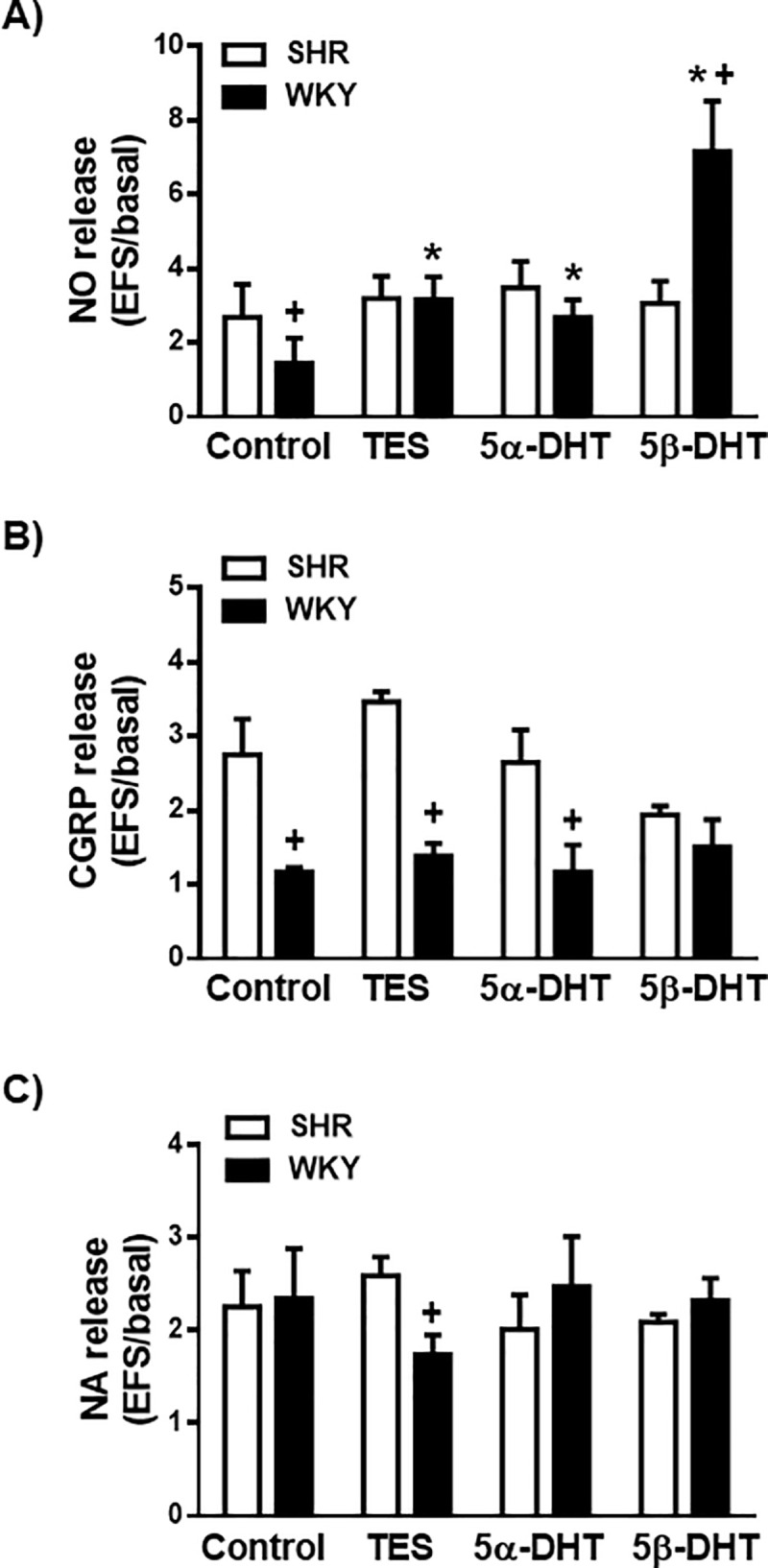
Effect of androgens on the neuronal NO, CGRP and NA release. Effect of 10 nM testosterone (TES), 5α-dihydrotestosterone (5α-DHT) and 5β- dihydrotestosterone (DHT) on the release of neuronal NO (A), CGRP (B) and NA (C) in the mesenteric artery of hypertensive (SHR) and normotensive (WKY) rats. Results (mean ± SEM) are expressed as the ratio between electrical field stimulation (EFS)-induced neurotransmitter release and basal release. Number of animals used, n = 4–5 animals in each group. *p <0.05 compared to control condition (vehicle group) in the respective rat strain; +*p* < 0.05 compared to SHR.

Incubation of mesenteric rings from SHR or WKY rats with 10 nM TES, 5α- or 5β-DHT did not significantly modify the basal NO and CGRP release (Table 1); the basal NA release was reduced by TES and 5β-DHT in arteries of SHR, whereas it was reduced only by 5β-DHT in arteries from WKY rats (Table 1). The EFS-induced NO, CGRP and NA release was similar in arteries from SHR or WKY rats in the presence or absence of ETOH (data not shown). EFS-induced NO release was reduced in the arteries from WKY rats in relation to the SHR (*p* < 0.01), in agreement with previous studies [25, 31]. Incubation of mesenteric arteries from SHR with 10 nM TES, 5α- or 5β-DHT did not significantly modify EFS-induced NO release (Fig 1). However, in mesenteric arteries from WKY rats, incubation with TES, 5α- or 5β-DHT resulted in an increase in the EFS-induced NO release (Fig 1A).

**Table 1:** Effect of 10 nM testosterone (TES), 5α-dihydrotestosterone (5α-DHT) and 5β-dihydrotestosterone (5β-DHT) on the basal release of NO, CGRP and NA in the mesenteric artery of spontaneously hypertensive (SHR) and normotensive (WKY) rats.

	NO		CGRP		NA	
	*SHR*	*WKY*	*SHR*	*WKY*	*SHR*	*WKY*
**Control**	1	1	1	1	1	1
**TES**	1.08 ± 0.1	0.97 ± 0.06	1.15 ± 0.06	1.06 ± 0.18	0.4 ± 0.07*	0.65 ± 0.09
**5α-DHT**	1.28 ± 0.14	0.84 ± 0.05	1.11 ± 0.09	0.89 ± 0.15	0.87 ± 0.1	0.78 ± 0.1
**5β-DHT**	0.96 ± 0.13	0.94 ± 0.1	0.94 ± 0.1	0.78 ± 0.03	0.46 ± 0.03*	0.46 ± 0.04*

Results (means ± SEM) are expressed relative to the release in control condition (vehicle group, ETOH) in the respective rat strain.

*p < 0.05 compared to control condition.

The EFS-induced CGRP release was greater in arteries of SHR than in those of the WKY rats (*p* < 0.05) (Fig 1B). In the arterial rings from the SHR or WKY rats, incubation with TES, 5α- or 5β-DHT did not significantly modify the EFS-induced CGRP (Fig 1B).

Regarding NA (Fig 1C), the EFS-induced NA release was similar in the arteries of both rat strains (*p* > 0.05). Incubation with TES, 5α- or 5β-DHT did not significantly modify EFS-induced NA release in the arteries of SHR or WKY rats (Fig 1C).

### Effect of androgens on vasomotor response to NO, CGRP and NA

**Fig 2:**
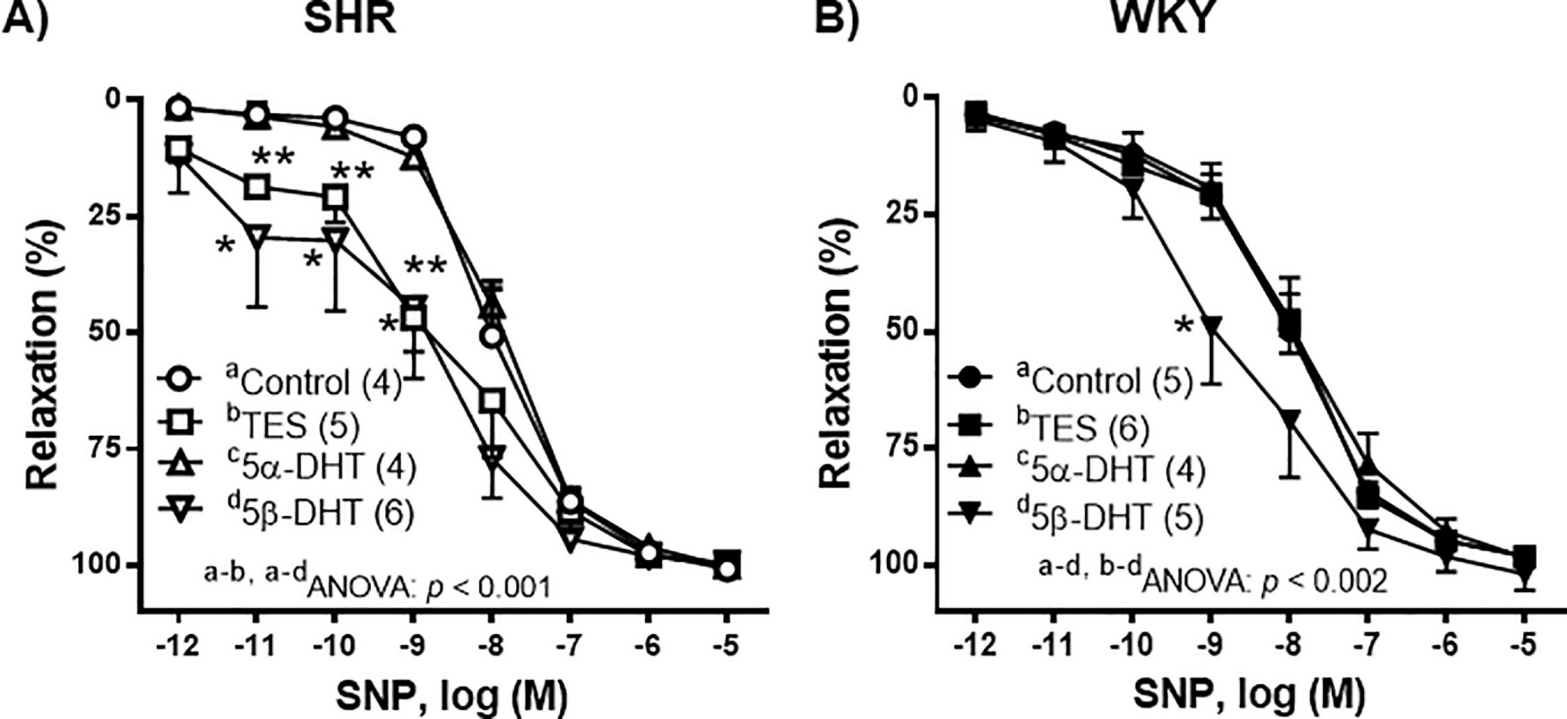
Effect of androgens on the vasodilator response to sodium nitroprusside (SNP). Effect of 10 nM testosterone (TES), 5α-dihydrotestosterone (5α-DHT) and 5β- dihydrotestosterone (DHT) on the concentration-response curves to sodium nitroprusside (SNP) in the mesenteric arteries of hypertensive (SHR) and normotensive (WKY) rats. Results (mean ± SEM) are expressed as the percentage of the inhibition of the contraction induced by 1 μM noradrenaline. *p < 0.01, **p < 0.001 compare to control condition (vehicle group). Number of animals used, n = 4–6.

To evaluate the effect of androgens on the vasomotor response induced by the released NO, SNP-induced responses were analyzed in the presence of each androgen. Incubation of arteries with 10 nM TES, 5α-DHT, 5β-DHT or with the equivalent volume of ETOH did not alter the basal vascular tone. In the NA-precontracted arteries from the control group (*i*.*e*., incubated with the vehicle), SNP (1 pM—10 μM) induced a concentration-dependent relaxation that was not statistically different between the arteries of SHR or WKY rats (*p* > 0.05). In the arterial segments from SHR, incubation with 10 nM 5α-DHT did not significantly modify SNP-induced relaxation, whereas incubation with 10 nM TES or 5β-DHT caused an increase in the response to SNP (Fig 2A). This increase was more notable at low SNP concentrations (1 pM—1 nM), particularly in the arteries incubated with 5β-DHT, where in three of the nine experiments 1 pM SNP induced a 60% relaxation rate (data not shown). In the arteries of WKY rats, incubation with TES or 5α-DHT did not significantly modify SNP-induced relaxation, whilst incubation with 5β-DHT increased the response induced by 1 nM SNP (Fig 2B).

**Fig 3:**
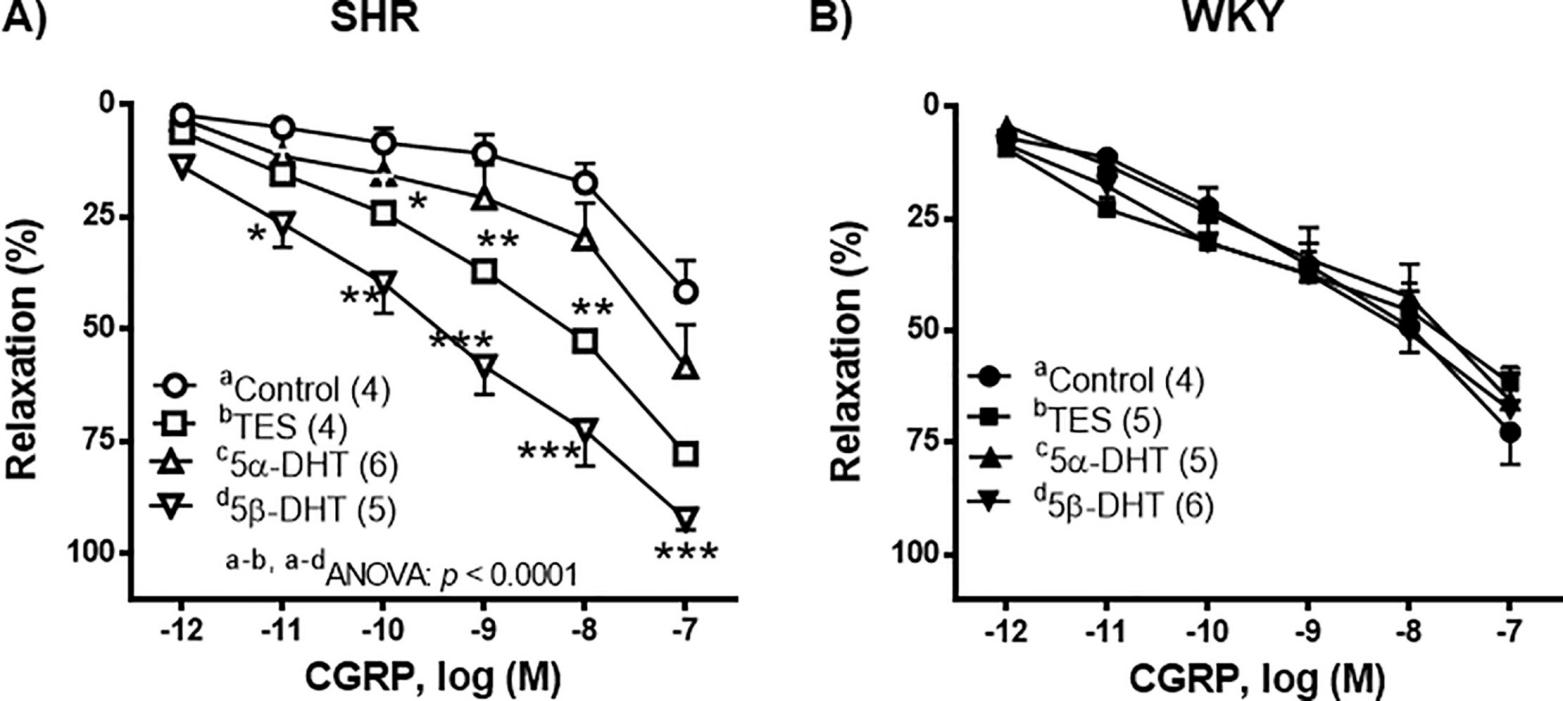
Effect of androgens on the vasodilator response to calcitonin gene-related peptide (CGRP). Effect of 10 nM testosterone (TES), 5α-dihydrotestosterone (5α-DHT) and 5β- dihydrotestosterone (DHT) on the concentration-response curves to calcitonin gene-related peptide (CGRP) in mesenteric arteries of hypertensive (SHR) and normotensive (WKY) rats. Results (mean ± SEM) are expressed as the percentage of the inhibition of the contraction induced by 1 μM noradrenaline. *p < 0.05, **p < 0.01, ***p < 0.001 compare to control condition (vehicle group). Number of animals used, n = 4–6.

In the NA-precontracted arteries, the vasodilator response induced by CGRP (1 pM—0.1 μM) was weaker in the arteries of SHR than in those of WKY rats (*p* < 0.05). Arteries from SHR incubated with TES or 5β-DHT showed a significant increase in the relaxation induced by CGRP (Fig 3A) while, in the mesenteric arteries from the WKY rats, androgens did not modify the CGRP-induced vasorelaxation (Fig 3B).

**Fig 4:**
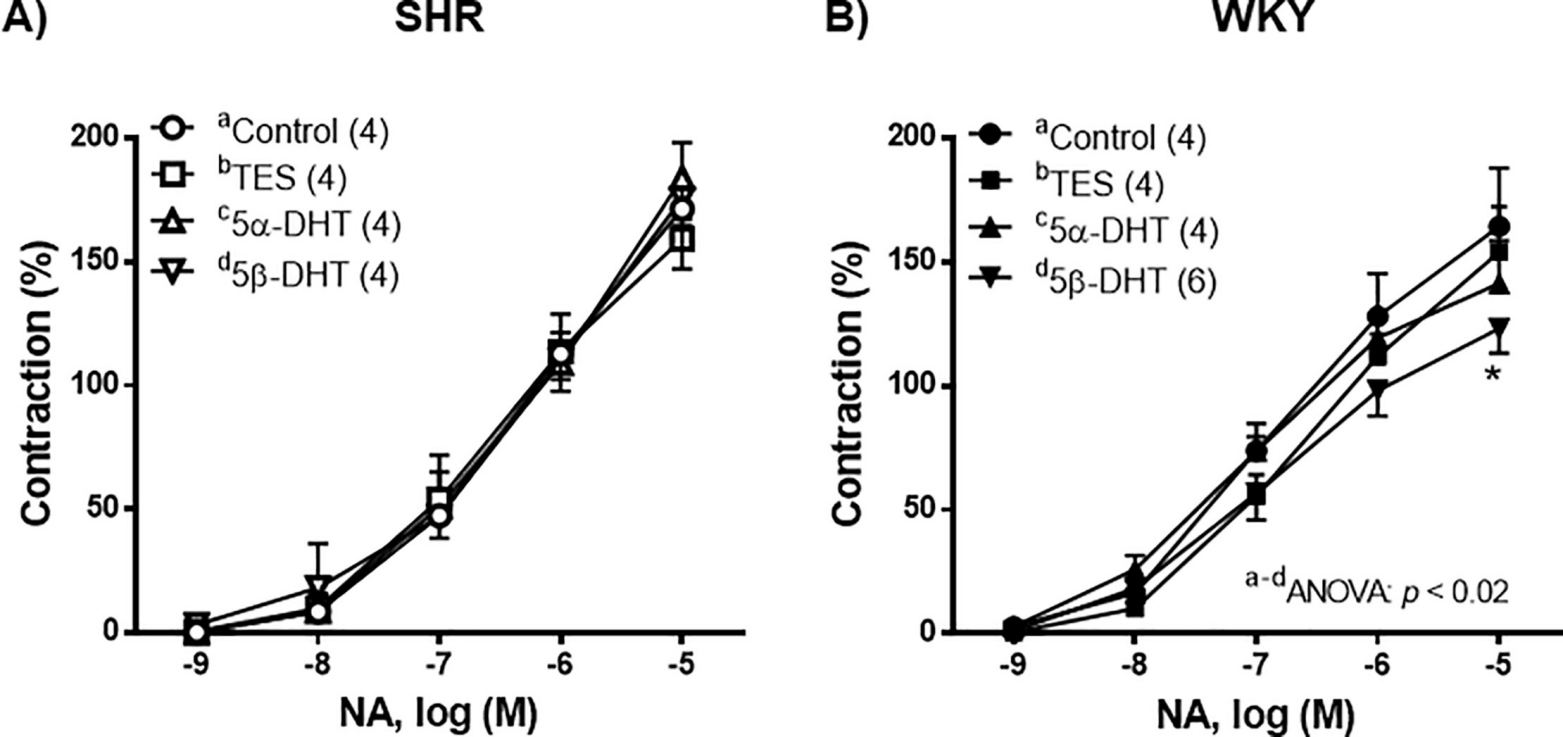
Effect of androgens on the contractile response to noradrenaline (NA). Effect of 10 nM testosterone (TES), 5α-dihydrotestosterone (5α-DHT) and 5β- dihydrotestosterone (DHT) on the concentration-response curves to noradrenaline (NA) in mesenteric arteries of hypertensive (SHR) and normotensive (WKY) rats. Results (mean ± SEM) are expressed as a percentage of the contraction induced by 60 mM KCl. *p <0.05 compare to control condition (vehicle group). Number of animals used, n = 4–6.

The contractile response induced by NA (1 nM—10 μM) was similar in the control arteries of both the SHR and the WKY rats (*p* > 0.05). In the arteries of the SHR, incubation with TES, 5α- or 5β-DHT did not significantly modify the contraction induced by NA in comparison with the control group (Fig 4A). In the arteries of WKY rats, incubation with TES or 5α-DHT did not alter the NA-induced response, whereas 5β-DHT caused a decrease in this response (Fig 4B).

## Discussion

The current study provides information on the effect of TES and of its dihydro 5-reduced metabolites (5α- and 5β-DHT) on the release and vasomotor function of the neurotransmitters NO, CGRP and NA in the mesenteric arteries of SHR and WKY rats. The results reveal that the modulatory action of androgens on the release/function of these neurotransmitters appears to differ depending on whether the arteries belong to SHR or WKY rats.

Rat mesenteric arteries possess rich sympathetic [32], sensory [33] and nitrergic [34] innervations, which are involved in the modulation of vasomotor tone. Alterations in the functionality of the perivascular innervation are known to play an important role in hypertension. In fact, hypertension has been reported to alter the release and/or function of neuronal NO [24, 25, 31], CGRP [26, 35] and NA [36–38].

Therefore, the current study showed that the EFS-induced NO release was significantly higher in arteries of SHR than in those of WKY rats. These results agree with those of previous studies [24, 25], suggesting that the increased neuronal NO release observed in the arteries of SHR may reflect a compensatory mechanism for the increased vascular resistance observed in hypertension. Regarding the effect of androgens on NO release, the results revealed that incubation of the mesenteric arteries of the SHR with TES, 5α- or 5β-DHT did not modify the EFS-induced NO release observed in the absence of androgens. However, in the arteries of WKY rats, incubation with each of the three androgens increased EFS-induced NO release. These results suggest that the mechanism by which androgens increase NO release in WKY rats does not appear to be functional in arteries from SHR. In this sense, TES and 5α-DHT have been described to activate PKC [39] which positively regulates endothelial NO synthase (eNOS) [28] and neuronal NO synthase (nNOS) [1, 25]. The fact that none of the androgens increased NO release could indicate that nNOS was already fully activated through PKC- and PKA-dependent mechanisms in the arteries of the SHR [24, 31] and that androgens were therefore incapable to further increase the activity of nNOS. On the contrary, in the arteries from normotensive rats, androgens appear to be able to activate nNOS since increased neuronal NO release in response to EFS was observed following the incubation of the androgens, particularly with 5β-DHT. These findings are consistent with the involvement of nNOS in the TES-induced hypotensive effect described in normotensive male Sprague-Dawley rats [40], but they are in contrast with the fact that the basal release of NO was not modified by any of the androgens. In this regard, endogenous androgens have been reported to modulate the basal NO release in a time-dependent manner [6]. Furthermore, these apparent discrepancies could reflect possible differences in the mechanisms working in *in vivo* and *in vitro* experimentation.

Concerning the release of CGRP, EFS produced a significant increase in mesenteric arteries from SHR, as previously reported [33, 41, 42]. However, in the arteries of WKY rats, the results showed that EFS failed to elevate the release of CGRP, a fact that is consistent with what has been described for mesenteric artery of normotensive rats [43]. Indeed, the CGRP released under physiological conditions does not appear to play an important role in the regulation of vascular tone, whereas in pathological conditions, such as hypertension, it exerts a cardiovascular protective effect [35]. It has been described that sex hormones can modify CGRP release, although the results are contradictory. Thus, the regulatory action seems to be time-dependent because the CGRP release from the rat mesenteric artery showed a significant increase after 1 month of orchidectomy, decreasing after 4 months of orchidectomy; these alterations were restored by testosterone replacement [44]. The current study demonstrated that incubation with TES, 5α- or 5β- DHT did not modify the EFS-induced CGRP release in the arteries of SHR or WKY rats. Nonetheless, their effects upon the vasodilatory response of CGRP cannot be ruled out.

With regard to NA release, the application of EFS to the mesenteric rings of SHR and WKY rats caused a significant increase in the release of NA, as reported for the mesenteric arteries of hypertensive [25] or normotensive [45] rats. Furthermore, the results revealed no difference in the EFS-induced NA release between arteries from SHR or WKY rats, as previously reported [30, 46]. However, an increase [36, 37] or a decrease [47] in NA release in the arteries of hypertensive rats in relation to normotensive ones has also been described. These discrepancies could be due to differences in experimental conditions, such as the age of the animals, which could induce functional modifications of some systems (*i*.*e*., angiotensin 1-7/Mas receptor) which, in turn, could modify NA release [48]. As for the effect of androgens on NA release, different results have been reported. Thus, both an increase [49, 50] and a decrease [51] in NA release induced by TES have been described. The results of our research have shown that, although incubation with any of the three androgens significantly modifies the EFS-induced NA release, the basal NA release was reduced by TES and 5β-DHT in the arteries of SHR, whereas in the arteries of WKY rats only 5β-DHT reduced that release; these actions could be contributing to the hypotensive/antihypertensive efficacy of 5β-DHT reported in conscious rats [7]. From the release experiments it can be concluded that androgens, particularly 5β-DHT, increased the EFS-induced NO release only in the arteries of WKY rats. Although another cellular mediator cannot be ruled out, a fully activated PKC in the arteries of SHR likely precludes a subsequent androgens activation and therefore an increase in the EFS-induced neurotransmitters release. The fact that 5β-DHT also reduced the basal NA release in the arteries of both SHR and WKY rats encourages further investigation. However, the next step in the current study was to analyze the effect of androgens on the vasomotor responses induced by the released neurotransmitters.

Many studies have linked the direct vasodilatory action of androgens in general, and 5β-DHT in particular, with the hypotensive/antihypertensive effects induced by these compounds in male [7, 8, 19, 40] and female [52] animal models. Therefore, the effects of androgens on the vasodilator response to NO and CGRP have been analyzed. In relation to the vasodilator response induced by NO, the SNP-induced responses were studied in the presence of TES, 5α- or 5β-DHT. Results showed that the SNP-induced vasodilation was similar in the arteries of SHR and WKY rats, as previously reported [47, 53]. In the arteries of SHR, incubation with TES or 5β-DHT increased the SNP-induced response, while 5α-DHT was found to have no effect. In the mesenteric arteries of WKY rats, only 5β-DHT caused an increase in the SNP-induced relaxation, although to a lesser extent than that observed in the SHR. It seems unlikely that the observed differences were due to differences in the antioxidant properties of androgens [54, 55]; rather, they would appear to result from the action of androgens on potassium channels, as reported for TES [56, 57], because the activation of potassium channels is also involved in the SNP-induced vasodilation [58]. Furthermore, as TES has been reported to increase cGMP [57] and cAMP [59] formation, the possible androgen-induced modifications of these second messengers should be taking into account in future studies.

With respect to the vasodilatation induced by CGRP, our results showed that the response was lower in the arteries of SHR than in WKY rats. However, other studies have reported similar [26] or stronger [60] vasodilator responses to CGRP in the arteries of SHR compared with those of WKY rats. These discrepancies may be due to differences in the experimental conditions, since de-endothelized arteries from SHR have been used in some analysis *versus* arteries with endothelium used in the present study. With regard to the effect of androgens on the vasodilator response to CGRP, our results showed that TES, and especially 5β-DHT, boosted this response in the arteries of SHR. Although it has been reported that female sex steroids caused an increase in the vasodilator response to CGRP in rat mesenteric arteries [61] by increasing the expression of different CGRP receptor components [62] and by activating potassium channels [63], there was no previous information on the effect of TES or 5β-DHT upon the response induced by CGRP in arteries of SHR.

The results of the study on the NA-induced contractions showed that no significant differences existed between the arteries of both strains of rats, as in previous studies [47, 64, 65]. However, some discrepancies exist, because a decrease [24, 66] or an increase [67, 68] in NA-induced responses in the arteries of SHR have been reported. Once again, variability in the age of the animals and/or in the experimental conditions could be responsible for the differences among the studies. In relation to the effect of androgens on the NA-induce contraction in arteries from gonadectomized animals, an increase [1], decrease [69] or no modification [70] has been reported. However, no studies comparatively analyze the acute *in vitro* effect of TES, 5α- or 5β- DHT on this response. Our results showed that TES, 5α- or 5β-DHT did not modify the NA-induced response in mesenteric arteries of SHR, whereas in the arteries of WKY rats, only 5β-DHT reduced the response to NA.

It is important to mention that although the experiments have been performed in the superior mesenteric artery, the results can be extended to the whole splanchnic arterial bed, since measurement of superior mesenteric arterial blood flow and resistance has been reported to indicate hemodynamic change in this arterial vascular bed [71, 72]. On the other hand, and despite that the effect of 10 nM androgens on blood pressure has not been analyzed in the current investigation, the effect of the three androgens (at the particular concentration of 10 nM) on the vasomotor responses described in the present study appears to be due to the androgens-induced modification in cell signaling pathways [57, 59, 63] rather than the androgens-induced hypotension. This assumption, based on that the acute administration of androgens, at micromolar concentrations, has been demonstrated to induce hypotension in conscious rats [7, 40, 52]; this action was related to the non-genomic androgens-induced vasorelaxation, since in vagosympathectomized pithed rats (in which the participation of the central and autonomic nervous systems was excluded) androgens were also capable of inducing hypotension [18]. Additionally, in the present study -unlike the previous ones-, a lower concentration of androgens was used, therefore, it would be expected that the hypotensive effect induced by 10 nM androgens would be less than those reported for the micromolar concentration range. Undoubtedly, these results raise considerations and open a series of future research that delves into the effects that androgens exert on the vascular function.

In summary, the results reveal that the modulatory effect of androgens differs between the arteries of SHR and WKY rats. The increase in the EFS-induced NO release and vasodilator action induced by 5β-DHT in the arteries of WKY rats, the increase in the CGRP- and SNP-induced vasodilatory response, especially by 5β-DHT, in the arteries of SHR and the reduction of the basal NA release might be contributing to the hypotensive/antihypertensive efficacy of 5β-DHT already described. In general terms, these results also support 5β-DHT (a genomically inactive metabolite lacking androgenic properties) as a possible therapeutic agent for the treatment of vascular pathologies such as hypertension.

## Supporting information

S1 Data Individual experimental data points for Fig 1A, 1B and 1C. (XLSX)

S2 Data Individual experimental data points for Fig 2A and Fig 2B. (XLSX)

S3 Data Individual experimental data points for Fig 3A and 3B. (XLSX)

S4 Data Individual experimental data points for Fig 4A and 4B. (XLSX)
